# Enhancing *Escherichia coli* Inactivation: Synergistic Mechanism of Ultraviolet Light and High-Voltage Electric Field

**DOI:** 10.3390/foods13091343

**Published:** 2024-04-26

**Authors:** Yihan Zhang, Yun Liang, Di Pan, Shupei Bai, Diya Wen, Min Tang, Hua Song, Xuan Guo, Hao Han

**Affiliations:** 1 School of Light Industry and Engineering, South China University of Technology, Guangzhou 510640, China; 202121029908@mail.scut.edu.cn (Y.Z.); liangyun@scut.edu.cn (Y.L.); 2 State Key Laboratory of NBC Protection for Civilian, Beijing 102205, China; pandiy@163.com (D.P.); baisp@263.net (S.B.); wdy-wdy2008@163.com (D.W.); xuan.guo@siat.ac.cn (X.G.); thinkinghh@163.com (H.H.)

**Keywords:** UVC, HVEF, synergistic effect, bactericidal mechanism

## Abstract

This study investigated the bactericidal effects of ultraviolet (UV) radiation, a high-voltage electric field (HVEF), and their combination on *Escherichia coli*. The results indicated that UV and combined disinfection were more effective with longer exposure, leading to significant reductions in microbial activity. Specifically, the single UV disinfection alone reduced activity by 3.3 log after 5 min, while combined disinfection achieved a 4.2 log reduction. In contrast, short-term HVEF treatment did not exhibit significant bactericidal effects, only achieving a reduction of 0.17 log in 5 min. Furthermore, prolonged exposure to both UV disinfection and an HVEF was found to damage cell membranes, ultimately causing cell death, while shorter durations did not. Despite rapid cell count decreases, flow cytometry did not detect apoptotic or necrotic cells, likely due to rapid cell rupture. This study suggests that combining UV radiation and an HVEF could be a promising approach for inhibiting bacterial reproduction, with HVEF enhancing UV effects. These findings provide insights for using combined HVEF and UV disinfection in food safety and preservation.

## 1. Introduction

Ultraviolet (UV) disinfection has been widely used for food sterilization, air purification, and water disinfection. The predominant method for sterilization involves the utilization of short-wave ultraviolet light in the range of 200–280 nm, commonly referred to as UVC. This cost-effective and easily accessible technique produces no harmful byproducts to human health [1]. The mechanism of action against bacteria involves disrupting their genetic material, resulting in the formation of covalent pyrimidine dimers between adenine molecules. This ultimately hinders bacterial replication and effectively renders them inactive [2]. The optimal wavelength for inactivating *E. coli* is approximately 265 nm [3]. Continuous exposure to 265 nm UV-LED light typically requires approximately 10 mJ/cm^2^ for a 2.5 log inactivation [4]. Although UV light can rapidly and effectively inactivate microorganisms [5], some organisms may undergo photoactivation, leading to incomplete sterilization [6]. Additionally, UV light has limited penetration capabilities and may struggle to eliminate microorganisms residing in hidden or shielded locations. Therefore, it is necessary to use hurdle technology to achieve comprehensive sterilization. Food preservation can be accomplished through a multitude of strategies, which encompass the reduction in water activity (aw), utilization of low-temperature storage, adjustment of pH levels, incorporation of competitive microflora, and the application of preservatives. The amalgamation of these distinct techniques is recognized as the “hurdle effect”, which serves to enhance the overall efficacy of the preservation process [7]. HVEF technology is a novel sterilization technology with low temperature and lower energy consumption [8,9]. The sterilization mechanism of this technology involves inducing electroporation in bacteria through the application of an electric field [10]. Previous research has highlighted the immense potential of an HVEF as a sterilization technique, showcasing its exceptional penetration capabilities, low energy consumption [11], cost-effective equipment, and an environmentally friendly and pollution-free sterilization process. Despite these advantages, the effectiveness of HVEFs in achieving complete sterilization remains somewhat limited [12].

Currently, there have been studies on combining an HVEF with UV disinfection for food sterilization. However, the electric fields used are mostly pulsed electric fields (PEFs), and the research targets are mainly liquid foods. Moreover, they only investigated the sterilization effect without delving into the underlying bactericidal mechanism [13,14,15]. 

Hence, the primary objective of this study is to delve into the distinct disinfection impacts of UV_265_ and HVEF, as well as to uncover the potential synergistic effects that arise from their combination. UV_265_ is known for its potent germicidal properties, albeit with restricted penetration capabilities. By combining UV_265_ with an HVEF, we aim to enhance its overall disinfection efficacy. Furthermore, this research seeks to unravel the intricate cellular-level mechanisms that underlie the disinfection effectiveness of this combined approach.

## 2. Materials and Methods

### 2.1. Agar Plate

First, 1.5% agar was melted by heating until it reached a liquid state. Subsequently, 39 mL of the liquefied agar was applied onto the surface of a stainless steel plate, ensuring its uniform distribution using a sterile applicator stick to achieve a thickness of 0.1 cm. After drying, 100 μL of bacterial suspension was evenly distributed and absorbed into 6 separate areas measuring 3 × 3 cm on the agar surface. The agar was then allowed to aerate and dry on a sterile operating table for 15 min.

### 2.2. Bacterial Strains

In this study, *Escherichia coli* (BN8099) was used as an indicator microorganism. A single colony of *E. coli* was selected and cultured in liquid lysogeny broth (LB) medium at 37 °C and 200 rpm for 12 h. The incubation process was repeated twice [16]. Subsequently, the bacteria were harvested by centrifugation at 8000 rpm for 5 min and then resuspended in phosphate-buffered saline (PBS). The process was repeated three times to obtain bacterial suspensions at a concentration of 10^8^ CFU/mL [17]. 

### 2.3. Disinfection Treatments

#### 2.3.1. Ultraviolet Treatment

The UV treatment was conducted using a low-pressure mercury lamp (Xingchuang Electronics Co. LTD, Guangzhou, China), and the light intensity was measured using a handheld UV light meter (Linshang Technology, Shenzhen, China). For this experiment, the UV intensity was adjusted to 30 μW/cm^2^ with a long emission wavelength of 265 nm and an output power of 5 W. The lamp was securely positioned at the center of a stainless steel plate measuring 20 × 20 cm. The irradiation time was controlled at 1, 3, and 5 min, and the UV lamp was turned on and allowed to stabilize for 15 min before the experiment.

#### 2.3.2. High-Voltage Electric Field Treatments

For this experiment, a negative high-voltage DC power supply (Dongwen high voltage, Tianjin, China) was utilized, capable of reaching a maximum output voltage of 50 kV. The wire-plate reactor dimensions were set at 20 × 20 × 20 cm, with three parallel wires serving as the high-voltage electrodes, and a stainless steel plate (19.8 × 19.8 × 0.3 cm) acting as the grounding electrode. The bactericidal efficacy of 12 kV, 10 kV, and 4 kV combined with UV irradiation was investigated. Among these voltages, the most consistent effect was observed at 10 kV; therefore, a voltage intensity of 10 kV was used in this study. The exposure times were controlled at intervals of 1, 3, and 5 min.

#### 2.3.3. Combined Treatment

**Figure 1:**
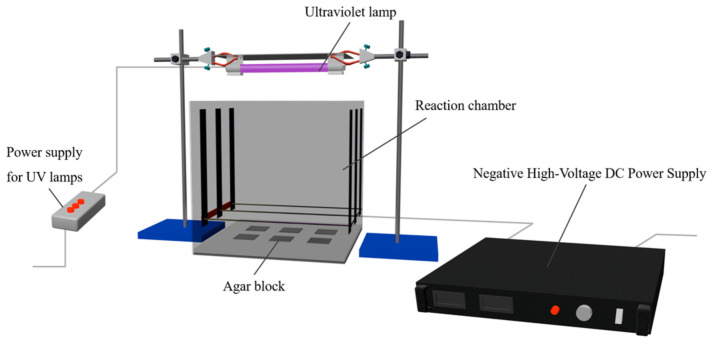
The schematic diagrams of the UV lamp, high-voltage power supply, and reaction chamber used in this experiment.

The experimental setup is depicted in Figure 1. The high-voltage power supply was activated, and the voltage was adjusted to the specified level. Concurrently, the ultraviolet lamp switch was turned on to create a synergistic inactivation effect on *E. coli* by subjecting them to combined exposure to ultraviolet radiation and a high-voltage electric field. The exposure times were controlled at intervals of 1, 3, and 5 min.

### 2.4. Characterization 

#### 2.4.1. Bactericidal Efficacy of UV, HVEF, and Combined Treatment on *E. coli*

The disinfection process was assessed using logarithmic decay analysis. The degree of inactivation was quantified as log(N_0_/N), where N_0_ and N represent the initial and final microbial counts, respectively. The viable bacterial count was determined using the plate counting method. Following disinfection, a 10-fold serial dilution of 100 µL of *E. coli* suspension was performed to ensure that the number of colonies on the final medium fell within the range of 30 to 300. Subsequently, 100 µL of the diluted sample was spread onto an agar solid medium for plate counting, which was then inverted and incubated at 37 °C for 24 h.

#### 2.4.2. The Integrity of Cell Membrane

##### Scanning Electron Microscopy (SEM)

The morphological changes and cellular damage resulting from various disinfection treatments were examined using an SEM. Following disinfection, the eluent containing *E. coli* was harvested and centrifuged at 8000 rpm for 5 min. The supernatant was discarded, and then 1 mL of 2.5% (vol/vol) glutaraldehyde was added to fix the cells overnight at 4 °C. Subsequently, the bacteria underwent three washes with PBS for 15 min each. After fixation with 1% osmic acid for 1.5 h, they were rinsed again. Dehydration was carried out by incrementally increasing the ethanol solution concentration (30–95%) before critical point drying, platinum coating, and observation under SEM (Hitachi SU3800, Tokyo, Japan).

##### Transmission Electron Microscopy (TEM)

The samples were subjected to dehydration in ethanol solutions with concentrations of 30%, 50%, 70%, and 90% for 10 min each, followed by exposure to pure ethanol and acetone for 15 min each. Subsequently, the samples were embedded at room temperature and cured in an oven. For staining purposes, the sections underwent treatment with a solution containing 2% uranyl acetate for 20 min, followed by lead citrate staining for 10 min. After drying, the stained cells were visualized using TEM (JEOL JEM-2100plus, Tokyo, Japan). 

##### Bicinchoninic Acid Assay

The bicinchoninic acid (BCA) assay was conducted to assess the amount of protein leakage after the disinfection treatment [18]. After undergoing UV irradiation, exposure to an electric field, and combined disinfection methods, the agar blocks were subjected to elution in 10 mL of PBS. Subsequently, 1 mL of the eluent was transferred into a tube and centrifuged at a speed of 8000 rpm for 5 min. The BCA assay was performed according to the protocol provided with the kit.

#### 2.4.3. The Permeability of Cell Membrane

##### Propidium Iodide Staining Assays

The sterilized agar blocks were eluted in 5 mL of PBS and centrifuged at 8000 rpm for 8 min. Then, 4 mL of supernatant was collected, followed by the addition of 20 μL of PI solution. The mixture was then incubated at 37 °C for 30 min. Subsequently, PI fluorescence photographs of various samples were captured using a microscope (SOPTOP, Ningbo, China).

#### 2.4.4. The Oxidative Damage of Cell Membrane

##### Reactive Oxygen Assay

The DCFH (2′,7′-dichlorodihydrofluorescein) probe is widely used for the detection of intracellular reactive oxygen (ROS) fluorescence. The prepared *E. coli* suspension was resuspended in a 10 μmol/L DCFH-DA solution and incubated at 37 °C for 20 min. Subsequently, the cells were washed 3 times with PBS to thoroughly eliminate any unbound probes. After the probes were loaded, the bactericidal treatment was administered, and the treated bacterial solution was collected. The fluorescence intensity of ROS in different samples was detected using a fluorescence spectrophotometer at the excitation wavelength of 488 nm and the emission wavelength of 525 nm. The relative fluorescence intensity was expressed as the ratio of the ROS fluorescence value of the treatment group to the corresponding bacterial survival logarithm, which is calculated by Formula (1).

(1) $$\mathrm{The}\;\mathrm{relative}\;\mathrm{fluorescence}\;\mathrm{intensity}=\frac{F}{\mathrm{lgN}}$$

F—the ROS fluorescence intensity;

N—the number of living bacteria.

#### 2.4.5. The Apoptosis of *E. coli*

For the apoptosis assay, the proportion of apoptotic cells was evaluated by dual staining with Annexin V-FITC and PI (Beyotime C1062, Shanghai, China). A combination of Annexin V-FITC and PI staining distinguished early apoptotic cells (Annexin V+, PI−) and late apoptotic cells (Annexin V+, PI+). The apoptosis of all stained cells was analyzed using a flow cytometer (BD Biosciences, San Jose, CA, USA).

#### 2.4.6. The Fluidity of the Cell Membrane

8-aniline-naphthalene-1-sulfonate (ANS) is an exogenous fluorescent probe extensively utilized for monitoring the conformational changes of biological macromolecules. The ANS solution, with a concentration of 8 mmol/L, was mixed with 4 mL of bactericidal eluent. The reaction took place at room temperature for 30 min, and the fluorescence intensity was measured using an excitation wavelength of 370 nm, an emission wavelength range of 400–600 nm, and a slit width of 10 nm.

### 2.5. Statistical Analysis 

Each experiment included three replicate samples, and each experiment was repeated a minimum of three times. The independent sample T-test was conducted using SPSS 29.0 SPSS (Inc., Chicago, IL, USA), with results of *p* < 0.01 (**) and *p* < 0.001 (***) considered statistically significant. All graphs were drawn using GraphPad Prism 9.5 (GraphPad Software, Inc., San Diego, CA, USA).

## 3. Results and Discussion

### 3.1. Inactivation of E. coli under Single or Combined Processes of UV and HVEF

**Figure 2:**
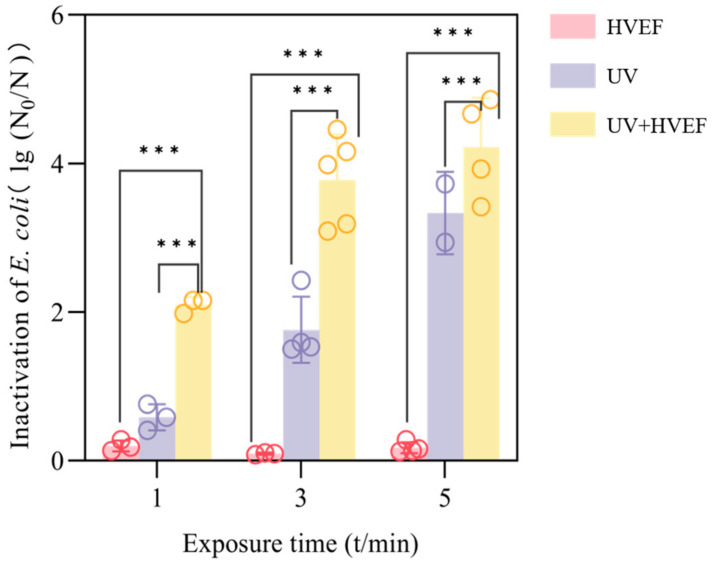
Inactivation characteristics of *E. coli* by single UV/HVEF and combined treatment. *p* < 0.001 (***).

It was observed that *E. coli* exhibited high sensitivity to UV light, as previous research described [1,19]. In this study, a 0.6 log reduction in *E. coli* was achieved by a dose of 1.8 mJ/cm^2^ (1 min exposure). When the UV dose reaches 9 mJ/cm^2^ (5 min), the inactivation of *E. coli* can achieve a reduction of 3.3 log. The combination of disinfection methods was shown to significantly enhance the efficacy of single UV disinfection, (*p* < 0.001), with a logarithmic reduction of 2.1 achieved within 1 min of combined action (Figure 2). Extending the duration to 3 min results in a bactericidal effect reaching 3.8 log (Figure 2), while a 5 min exposure achieves a bactericidal effect of 4.2 log (Figure 2). However, the enhancement in bactericidal efficacy becomes less pronounced at this stage. The reduction in *E. coli* under short-time HVEF stimulation is less than 1 log (Figure 2). The factors affecting electric field inactivation include electric field intensity and action time [20]. The limited inactivation effect may be attributed to the low electric field intensity and short action time. Fortunately, the synergistic effect of UV and HVEF treatment significantly enhances the bactericidal efficacy beyond that achieved by the HVEF alone at any given time, (*p* < 0.001). This could be due to the potent inactivation effect of UV light and its positive influence on electric field inactivation.

### 3.2. The Integrity of the Cell Membrane

#### 3.2.1. SEM

**Figure 3:**
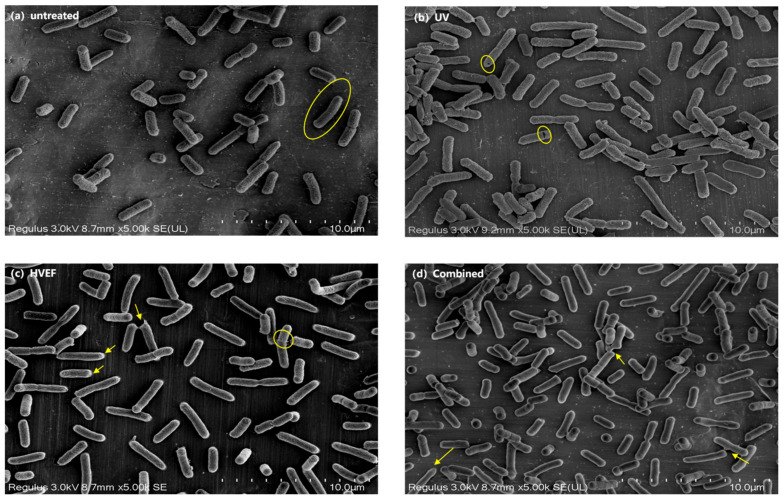
The SEM images of *E. coli* in the untreated, UV, HVEF, and combined treatments. The morphological changes to *E. coli* are circled in yellow; the damaged cell surface of *E. coli*, or leakage from cytoplasm, are marked by yellow arrows.

To determine the effect of HVEF, UV, and combined UV and HVEF treatment on cell morphology, including the permeability and integrity of cell membranes, changes in cell morphology were observed by SEM. The untreated *E. coli*, as shown in Figure 3a, exhibits a well-defined and uniform rod shape with distinct boundaries [21]. After exposure to UV light for 1 min, the *E. coli* exhibited significant damage to the head, and the surface displayed visible cracks (Figure 3b). The *E. coli* subjected to an HVEF treatment for 1 min exhibited the formation of cracks and a blurring of the edges [22], while still maintaining an intact cell membrane at this stage. This observation suggests that the applied voltage might not have been sufficient to induce cleavage of the cell membrane. Similar damage was found in *E. coli* treated by both UV light and the HVEF (Figure 3d).

#### 3.2.2. TEM

**Figure 4:**
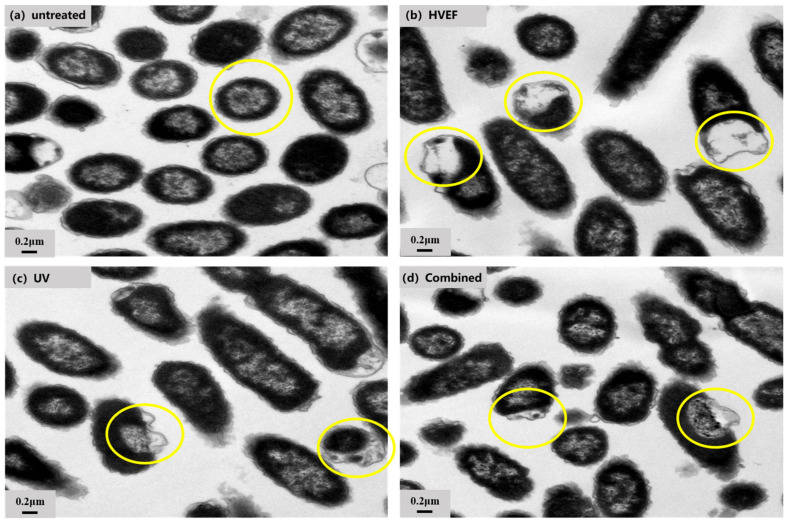
The TEM images of *E. coli* in the untreated, UV, HVEF, and combined treatments. The cytoplasmic changes in *E. coli* are circled in yellow.

The changes in cellular structure caused by HVEF, UV, and a combination of UV and HVEF treatment were observed by TEM. The results showed that the untreated *E. coli* cells were plump, with intact and transparent protoplasts (Figure 4a). Conversely, treated *E. coli* cells displayed noticeable alterations such as protoplast aggregation, loss, and plasma wall separation upon exposure to either singular UV or HVEF treatment (Figure 4b,c), as well as when subjected to combined treatment (Figure 4d).

#### 3.2.3. Protein Leakage

**Figure 5:**
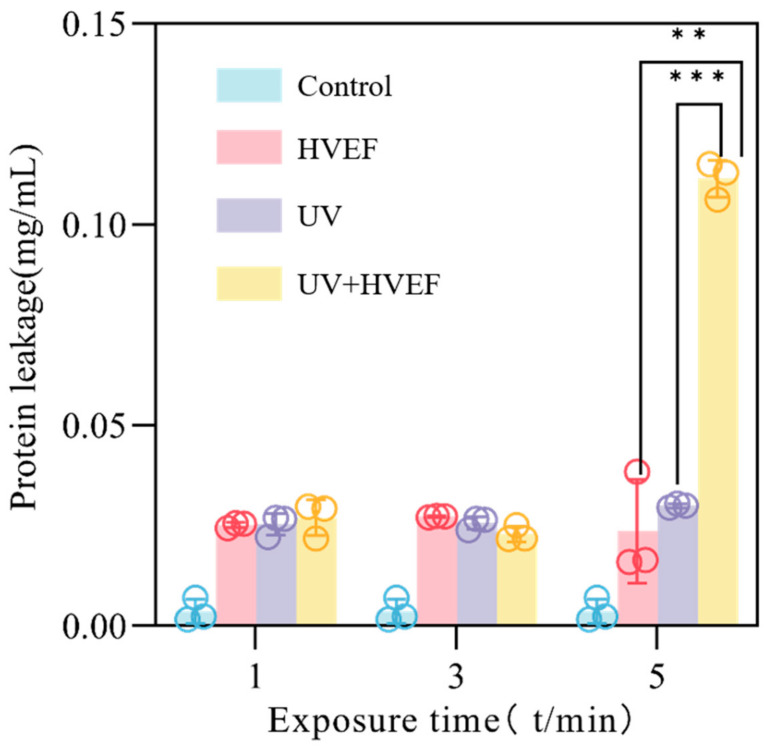
The protein leakage of *E. coli* before and after treatment with UV, HVEF, and their combination. *p* < 0.01 (**) and *p* < 0.001 (***).

In the case of UV disinfection, the level of protein leakage showed minimal variation over time, likely due to the primary destruction of cellular DNA by UV disinfection, with lesser damage to the cell membrane. For combined disinfection, there was no significant change in protein leakage within the first three minutes. However, after 5 min of treatment, there was a sharp increase in protein leakage (Figure 5), suggesting that the cell membrane may have reached its breaking point. This suggests that the combined treatment for 5 min may result in irreversible damage to the cell membrane of *E. coli*, leading to the leakage of intracellular proteins. Chen [23] also indicates that only when the concentration of vanillin reaches the MIC or above, it may cause irreversible damage to the cell membrane of *E. coli*. When the cell is exposed to an electric field, the charged substances within the cell undergo directional movement under the influence of this field. Within a short time, these charged substances redistribute themselves on either side of the cell membrane, creating a microelectronic field. The difference in potential between this microelectronic field is referred to as transmembrane potential (TMP). As the strength of the electric field increases or processing time extends, the polarization of the cell membrane intensifies and consequently enhances the strength of the newly formed microelectronic field. This leads to mutual attraction forces between hetero-ionic ions on both sides of the membrane, resulting in extrusion pressure exerted on each side. Consequently, the TMP continues to rise, causing a reduction in cell membrane thickness [10]. At this stage, the viscoelastic restoring force within the cell membrane initiates a response against the compressive forces from both directions. However, when the growth rate exceeds that of the viscoelastic restoring force, progressive thinning of the cell membrane occurs. Upon reaching a transmembrane potential of 1 V, partial damage is experienced by the cell membrane, followed by an intensification of the electric field strength leading to larger perforations and irreversible breakdown, ultimately resulting in cell death [24].

### 3.3. The Permeability of Cell Membrane

#### Propidium Iodinated Fluorescence

**Figure 6:**
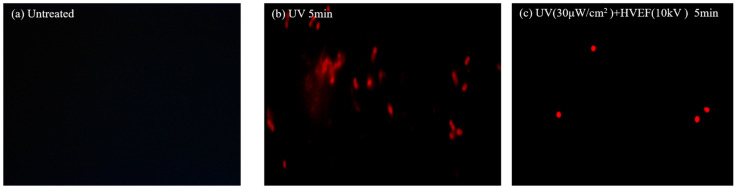
Fluorescence images of PI staining: (**a**) fluorescence images stained by PI of the untreated group; (**b**) fluorescence images stained by PI after 5 min of UV treatment; (**c**) fluorescence images stained by PI after combined UV and HVEF treatment for 5 min.

*Propidium iodide* (PI) is a commonly used fluorescent dye for DNA labeling, capable of selective and quantitative incorporation into the helical structure of both DNA and RNA. PI has limited solubility in membranes, which prevents its entry into intact bacterial cells. However, upon membrane damage, PI can permeate through the compromised cell membrane and specifically bind to nucleic acids, resulting in fluorescence emission [25]. Within the initial 3 min of the experiment, the *E. coli* cells in almost all treatments were as unstained as those in the control group (Figure 6a); however, after 5 min of treatment, both the UV treatment group and combined treatment group displayed red fluorescence (Figure 6b,c), indicating compromised cell membranes in *E. coli* resulting in nucleic acid leakage and subsequent binding with PI. The ability of UV radiation to penetrate the cell membrane and directly interact with cellular DNA may explain why, during the initial stages of UV disinfection, bacteria still maintain their intact cellular structure, preventing PI from entering the cell interior and binding to DNA. Similarly, electric field disinfection alone fails to break the cell membrane due to insufficient stimulation time despite a voltage of 10 kV being applied. However, when UV and HVEF are combined for 5 min, certain bacterial cell membranes begin to rupture, allowing for PI to enter the cells and bind with DNA, resulting in observable red fluorescence. Gan [26] utilized low-temperature plasma to inactivate *Escherichia coli* and *Saccharomyces cerevisiae* in chokeberry juice, and similar phenomena were observed in their research findings. Following plasma treatment, the cell membrane potential decreased, making the cell membrane susceptible to damage by active particles in the plasma, such as ROS. Subsequently, the lipid bilayer of the cell membrane was oxidized, leading to the leakage of cellular components from the cell and ultimately resulting in microbial cell death.

### 3.4. The Oxidative Damage of Cell Membrane

**Figure 7:**
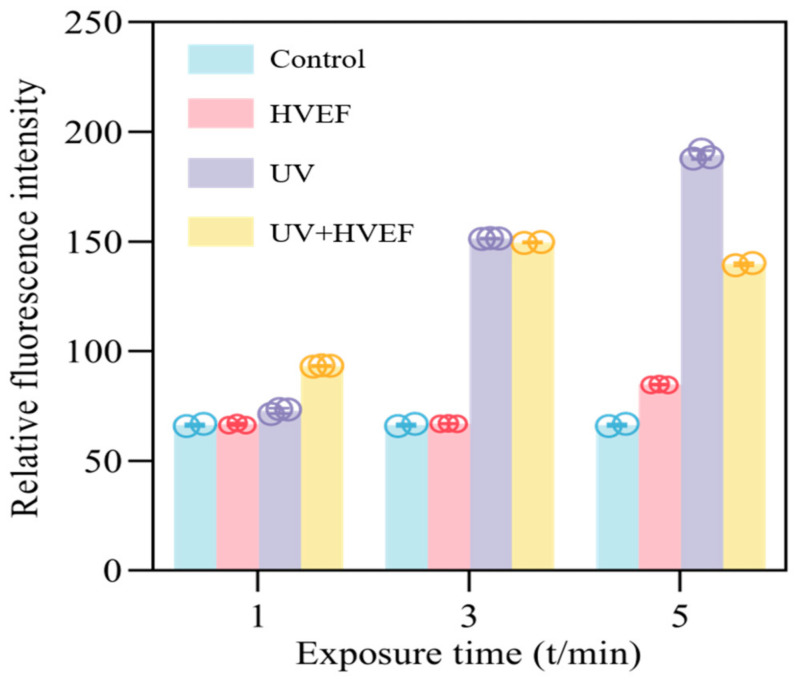
The relative ROS fluorescence intensity of *E. coli* before and after UV, HVEF, and their combination.

Reactive oxygen species (ROS) encompass oxygen-free radicals and certain non-free radicals that act as oxidants and can easily convert into free radicals [27]. The levels of reactive oxygen species (ROS) exhibit an instantaneous increase upon cellular stimulation by external factors [28]. If the production of ROS exceeds the antioxidant defense capacity, it can lead to cellular damage. The DCFH-DA molecule itself lacks fluorescence and exhibits the ability to freely traverse the cellular membrane. Once inside the cell, intracellular esterase breaks it down into DCFH, which cannot cross the cell membrane. Subsequently, intracellular ROS can further oxidize DCFH into highly fluorescent 2′,7′-dichlorodihydrofluorescein (DCF). The level of DCF content can be considered as directly proportional to the concentration of ROS formation [29]. In the untreated group, fluorescence intensity was also observed, attributed to continuous endogenous reactive oxygen species (ROS) production by bacterial cells during normal aerobic respiration [30]. Previous studies have shown that exposure to HVEF leads to a dramatic increase in the production of various ROS and oxidative stress [31,32]. Similarly, it has been shown that UV light induces the production of reactive oxygen species, which kills bacteria by moving towards the shielded area [33]. For *E. coli* treated solely with either HVEF or UV treatment, intracellular ROS exhibited an increasing trend as treatment time was prolonged, indicating that extending the duration of treatment indeed facilitated ROS accumulation. Throughout this period, the cell membrane remained intact, leading to a gradual increase in fluorescence intensity. However, when UV and HVEF disinfection were combined, the fluorescence intensity increased within 1–3 min but decreased after 5 min of treatment. This observed decrease could be attributed to the rupture of the cell membrane following a combined treatment for 5 min, leading to the release of cellular components including ROS (Figure 7). The aforementioned protein leakage results support this conclusion (Figure 5). Additionally, exogenous ROS accumulation within cells can disrupt various cellular contents such as proteins and nucleic acids, ultimately causing the deactivation of esterase protein that can no longer catalyze DCFH-DA into DCFH, henceforth leading to a decrease in fluorescence intensity [34]. These findings demonstrate that both UV and HVEF treatments induce exogenous ROS accumulation within *E. coli* cells, which subsequently attack biological macromolecules present inside these bacterial cells.

### 3.5. The Apoptosis of E. coli

**Figure 8:**
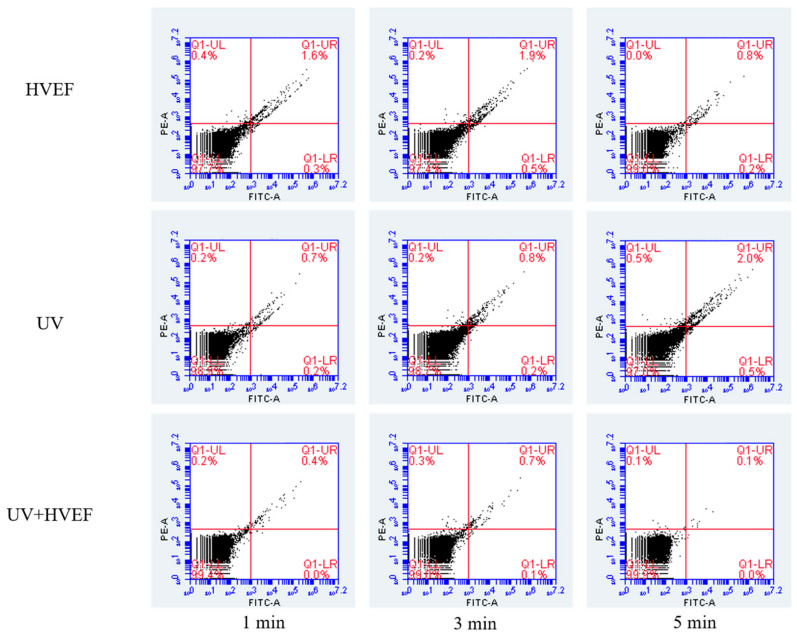
The apoptosis of *E. coli* cells before and after UV, HVEF, and their combination.

Annexin V possesses selective binding affinity for *phosphatidylserine*, which is predominantly located on the inner side of the cell membrane adjacent to the cytoplasm. During the early stages of apoptosis, various types of cells translocate *phosphatidylserine* to the outer surface of the cell membrane. Annexin V has a preference for negatively charged phospholipids and can be used in conjunction with luciferase to identify apoptotic cells. By staining cells with annexin V-FITC and *propidium iodide* (PI), it is possible to differentiate between viable normal, viable apoptotic, and apoptotic/necrotic cells simultaneously before plasma membrane permeability changes occur [35]. The experimental results are depicted in the figure, where the four quadrants *Q1-UL*, *Q1-UR*, *Q1-LL*, and *Q1-LR* correspondingly represent necrotic cells, early apoptotic cells, viable cells, and late apoptotic cells. The proportion of viable cells in the treated bacteria exceeds 97%, while the proportions of necrotic cells, early apoptotic cells, and late apoptotic cells remain below 1%. However, based on plate counting, it was observed that the population of *E. coli* exhibited a reduction of 3.3 log solely under UV exposure and a decrease of 4.2 log when subjected to the combined UV and HVEF disinfection, as depicted in Figure 8. This phenomenon may be attributed to the rapid cleavage of bacteria upon exposure to UV and HVEF stimulation resulting in a reduction in bacterial count; however, it is noteworthy that the proportion of viable cells remains significantly high. The bacteria subjected to electric field treatment exhibited a subtle alteration in the *Q1-UR* region, displaying an initial increase followed by a decrease. This observed trend could potentially be attributed to the progressive accumulation of damage effects, ultimately culminating in cellular demise. Zhao [36] investigated the impact of pulsed electric field (PEF) treatment on *Staphylococcus aureus*, *Escherichia coli*, and *Listeria monocytogenes* in milk. Their findings revealed a biphasic response in the sublethal fraction of bacterial cells, initially increasing before subsequently decreasing.

### 3.6. The Fluidity of Cell

**Figure 9:**
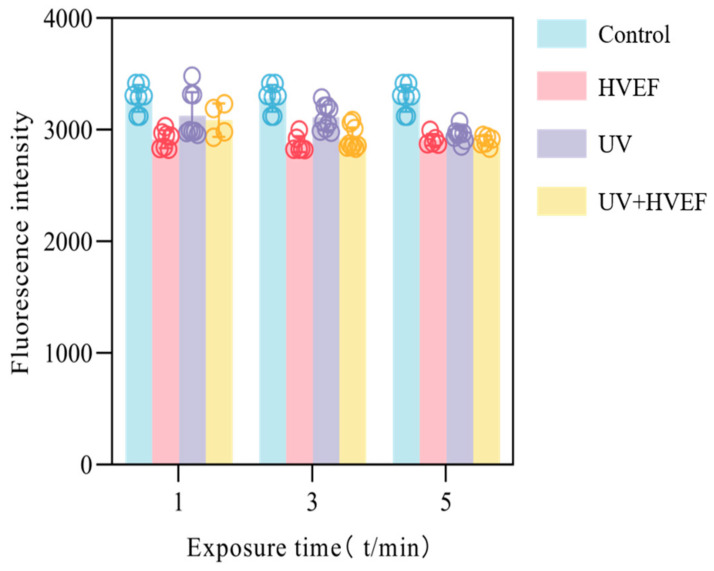
The ANS fluorescence intensity of *E. coli* before and after UV, HVEF, and their combination.

ANS exhibits only a faint green fluorescence in its free form when dissolved in an aqueous solution. However, upon binding to the hydrophobic region of the protein, it induces an intense blue fluorescence [34]. The ANS fluorescence intensity of *E. coli* is depicted in Figure 9, showcasing the effects of different treatments: untreated group, UV treatment, HVEF treatment, and combined treatment. All the treated groups’ fluorescence values were found to be lower compared to those of the untreated group, indicating that the three disinfection methods had altered the fluidity of the cell membrane. Moreover, it was observed that the UV treatment group exhibited higher fluorescence values in comparison to both the HVEF and combined treatment groups. This difference may be attributed to changes in cell membrane fluidity induced by HVEF stimulation, while UV primarily affects DNA within the cell without significantly impacting cell membrane fluidity. 

## 4. Conclusions

Our study demonstrates that a UV dose of 9 mJ/cm^2^ can achieve a 3.3 log reduction in microbial activity. The combined treatment of UV and HVEF surpasses the individual effects of UV or HVEF treatment alone, resulting in a remarkable 4.2 log reduction in disinfection within just 5 min. Therefore, the combination of UV and HVEF as a dual-disinfection method has emerged as a promising approach in the realm of microbial control, demonstrating a significant enhancement in disinfection efficacy compared to single-method treatments This innovative combination leverages the individual strengths of both UV and HVEF treatments to achieve a more comprehensive and effective elimination of microorganisms, thereby ensuring a higher standard of disinfection. The results of this study show that UV inactivates microbes by damaging their DNA, while HVEF eradicates bacteria by damaging their cell membranes. When these two technologies are combined, the synergistic effect is remarkable. The UV light first damages the microbial DNA, making the cells more susceptible to the effects of the HVEF. This combination not only increases the overall disinfection efficiency but also reduces the required intensity or duration of each treatment, thereby lowering energy consumption and operational costs. In conclusion, although the combination of UV and HVEF demonstrates potential in a controlled laboratory environment, its implementation in the food industry necessitates meticulous consideration. Thorough testing is imperative to guarantee the safety and efficacy of combined disinfection methods in food applications. This involves assessing their impact on various types of microorganisms commonly present in food, evaluating effects on key food quality attributes such as flavor, texture, and nutrient content, and determining the optimal treatment conditions to strike a balance between efficiency and preservation of food quality.

Furthermore, the integration of this technology into food production settings demands meticulous planning. Equipment and processes must be meticulously designed to ensure consistent exposure of food items to UV and HVEF treatment. This may necessitate the development of specialized equipment that can seamlessly integrate into existing food processing lines, along with the establishment of stringent operating protocols and quality assurance measures.

## Data Availability

The data presented in this study can be obtained upon request from the corresponding authors. These data will not be publicly disclosed due to privacy restrictions.
